# New Medical Journal

**Published:** 1860-01

**Authors:** 


*New Medical Journal.*—We are informed that a new medical journal will soon be issued in Cincinnati, under the editorial management of Professors Lawson and Blackman.

The character and qualifications of these gentlemen are a guarantee that it will be worthy of the best portion of the profession in Cincinnati, which has hitherto been greatly in need of a suitable organ.—*Chicago Med. Journal.*

